# Supplementary material for: Elevated MACC1 Expression in Colorectal Cancer Is Driven by Chromosomal Instability and Is Associated with Molecular Subtype and Worse Patient Survival
Source: Cancers (Basel). 2022 Mar 29;14(7):1749. doi: 10.3390/cancers14071749 (PMC8997143; doi:10.3390/cancers14071749)
Supplement: Supplementary file 1 [file cancers-14-01749-s001.zip › cancers-1627290-FC -supple_AM1.pdf]

cancers

MDPI

Supplemantary Materials:

Elevated MACC1 Expression in Colorectal Cancer Is Driven by Chromosomal Instability and Is Associated with Molecular Subtype and Worse Patient Survival

Vincent Vuaroqueaux, Alexandra Musch, Dennis Kobelt, Thomas Risch, Pia Herrmann, Susen Burock, Anne-Lise Peille, Marie-Laure Yaspo, Heinz-Herbert Fiebig and Ulrike Stein

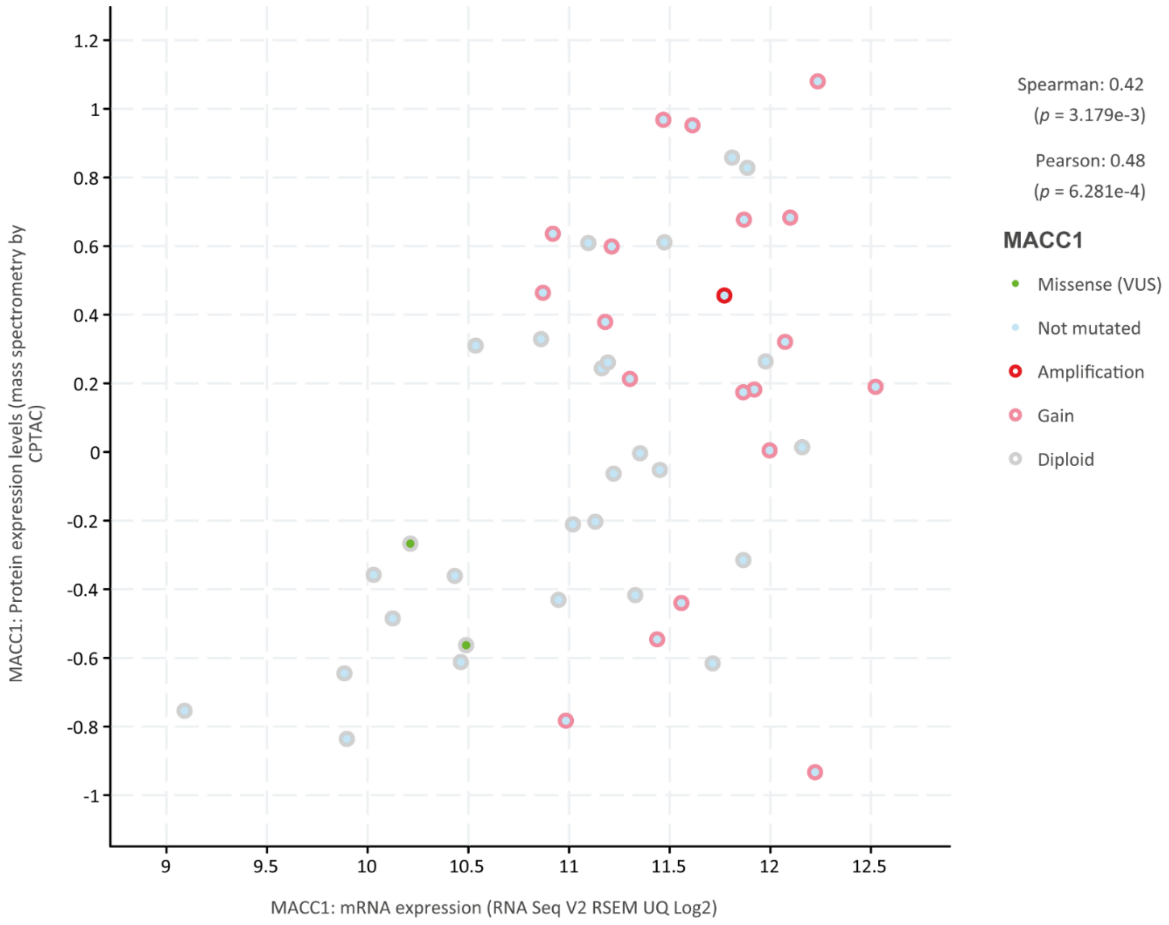

Figure S1. Scatter plot showing the correlaton between MACC1 mRNA and protein levels in CRC patient tumors. MACC1 mRNA levels were determined by RNA-Seq (data RSEM normalized – log2 transformed) and protein levels were determined by using mass spectrometry. MACC1 CNA and mutation status of the samples are represented by coloured dots The data are issued from a published CRC patient cohort (*N* = 48) (DOI:https://doi.org/10.1016/j.cell.2019.03.030). Data were obtained from https://www.cbioportal.org/ (accessed on 08.03.2022).

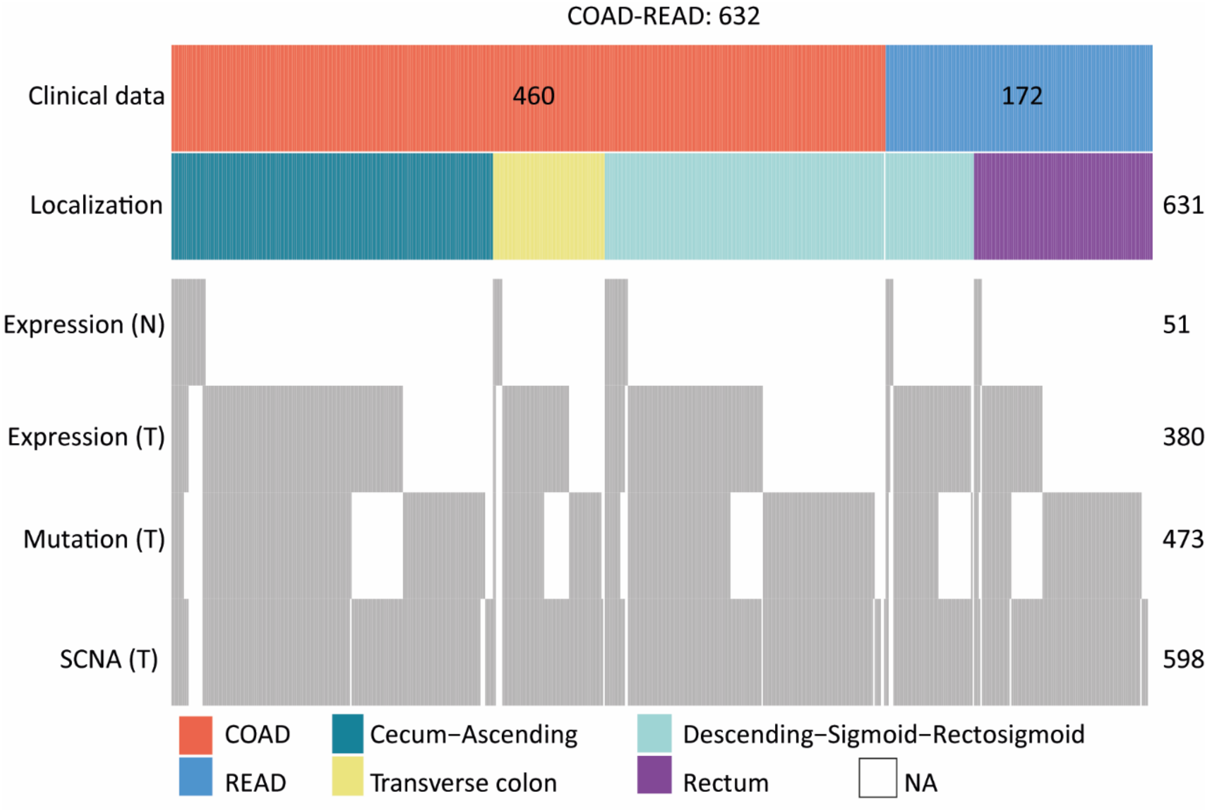

Figure S2. Visual representation of the number of samples and type of data in the analyzed COAD-READ TCGA2 dataset.

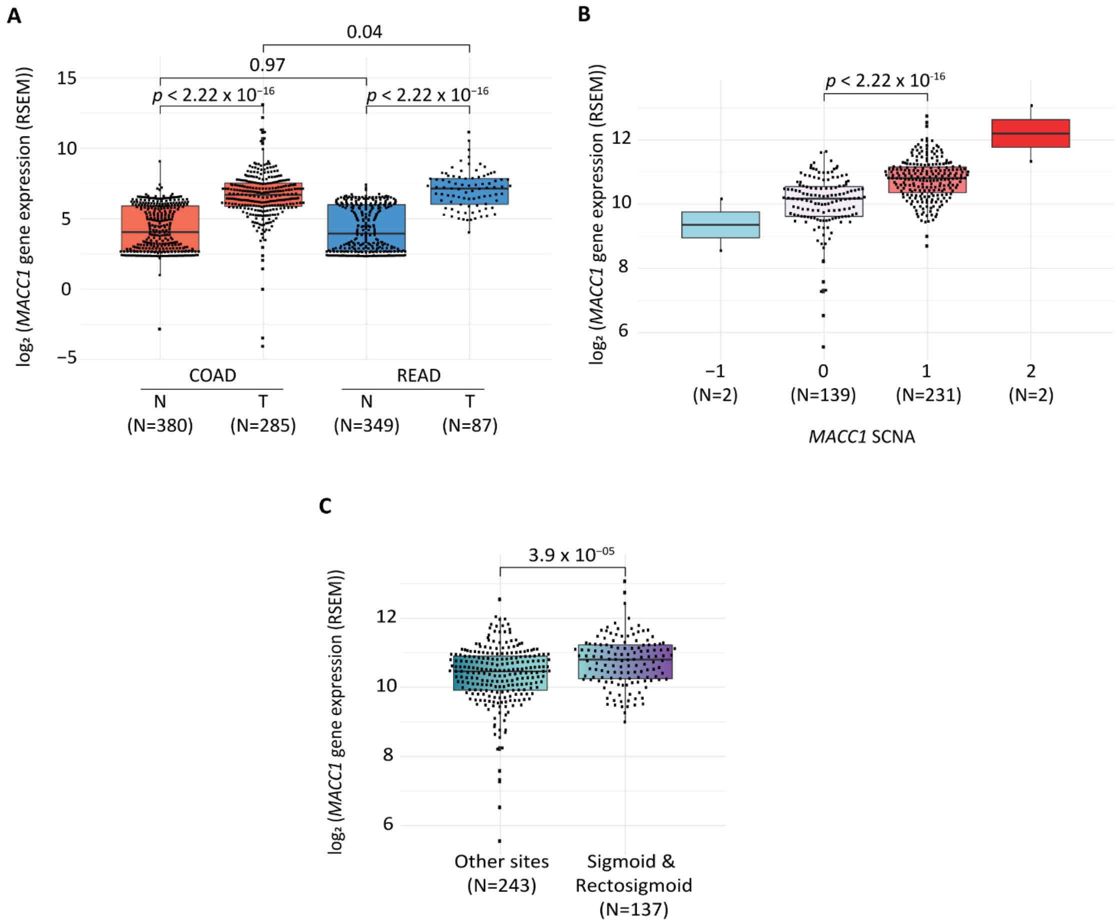

Figure S3. Statistical association of MACC1 SCNA and expression levels with CRC sample characteristics. A, Box plot showing MACC1 mRNA expression in normal and tumor tissues from the Wang et al. cohort of unified TCGA-GTEx colorectal samples [38]. B, MACC1 mRNA expression by MACC1 SCNA categories in the COAD-READ TCGA cohort. Horizontal bars indicate median values, boxes are interquartile range, whiskers indicate values within 1.5 times interquartile range. C, Box plot showing MACC1 mRNA expression differences in tumors localized in sigmoid and rectosigmoid parts compared to the other anatomic sites (ascending colon, cecum, descending colon, transverse colon, and rectum) in the COAD-8 READ TCGA cohort. A and B, horizontal bars indicate median values, boxes are interquartile range, whiskers indicate values within 1.5 times interquartile range.

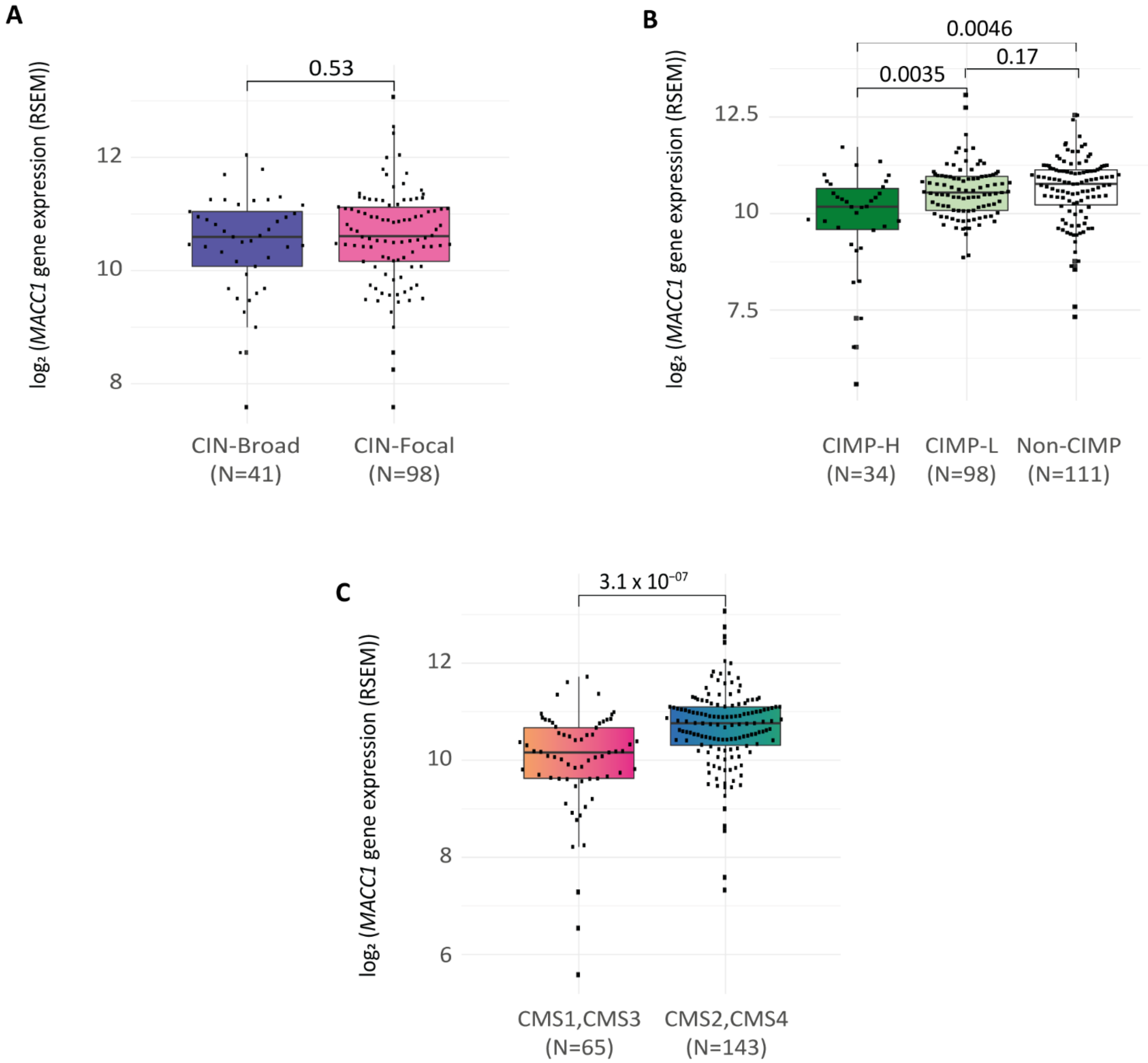

Figure S4. MACC1 mRNA expression and CIN, CIMP and CMS molecular subtypes in the COAD-READ TCGA cohort. A, Box plot showing MACC1 mRNA expression and CIN subgroups (CIN-Focal and CIN-Broad). B, Box plot showing MACC1 mRNA expression and tumors CpG island methylator phenotype (CIMP) status. C, Box plot showing MACC1 mRNA expression differences between CMS1-CMS3 and CMS2-CMS4 subtypes. A, B and C, horizontal bars indicate median values, boxes are interquartile range, whiskers indicate values within 1.5 times interquartile range.

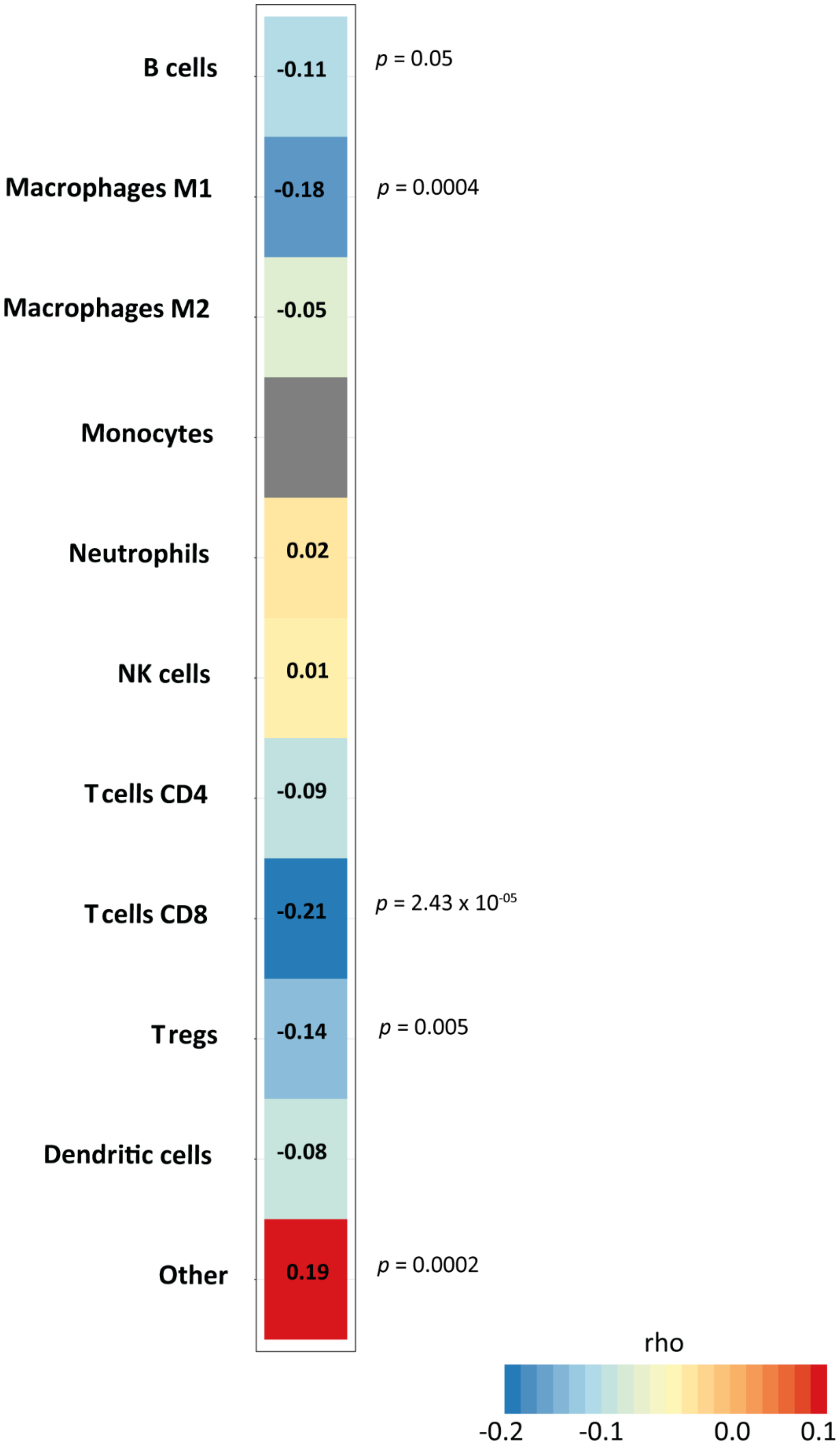

Figure S5. MACC1 mRNA expression and correlation with immune contexture of the tumors. A heatmap showing Spearman rho values between MACC1 mRNA expression levels and the proportion of different immune cell types estimated by quanTIseq in COAD-READ samples.

Table S1. Overview chart of the cohorts and the different methods used in the study.

Main cohorts	Analysis	Data type	Source	Identifier
OncoTrack (N=90)	Correlation of *MACC1* SCNA and mRNA (I)	*MACC1* SCNA (whole genome/exome sequencing) and mRNA (RNA-Seq)	Schütte M et al. [24]	DOI: 10.1038/ncomms14262
Charité metastasis cohort (N=35)	*MACC1* SCNA and CRC patient MFS	MACC1 SCNA (ddPCR) + clinical data	Internal data	
TCGA COAD-READ (N=632)	Correlation of *MACC1* SCNA and mRNA (II); association with clinical variables	*MACC1* SCNA (SNP6-GISTIC) + mRNA (RNA-Seq); + clinical data	Firehose/Broad institute	https://gdac.broadinstitute.org/
**Supplementary or derived cohorts**				
CPTAC-2 prospective (N=48)	Correlation *MACC1* protein and mRNA	mRNA (RNA-Seq), protein (mass spectrometry)	Vasaikar et al. [30]	DOI: 10.1016/j.cell.2019.03.030
COAD-READ/GTEx (N=1,101)	*MACC1* mRNA overexpression in CRC tumors compared with normal tissues	mRNA (RNA-Seq) of COAD-READ tumors unified with normal tissues from the GTEx database	Wang et al. [27]	DOI: 10.1038/sdata.2018.61
Liu Y et . al (COAD-READ subset) (N=243)	*MACC1* mRNA and CRC molecular subtypes	*MACC1* mRNA (RNA-Seq); + CRC molecular subtypes	Liu Y et al. [28]	DOI: 10.1016/j.ccell.2018.03.010

Table S2. Patient characteristics of the Charité metastasis cohort (N=35).

**UICC stage.**	**I**	**II**	**III**	
N (%)	9 (26%)	17 (48%)	9 (26%)	
**Sex**	**Male**	**Female**		
N (%)	19 (54%)	16 (46%)		
**Age group at diagnosis, years**	**30-50**	**51-60**	**61-70**	**>70**
N (%)	5 (14%)	7 (20%)	14 (40%)	9 (26%)
**Localization**	**Right colon**	**Left colon**	**Rectum**	
N (%)	12 (34%)	12 (34%)	11 (32%)	
**Metachronous metastasis**	**No**	**Yes**		
N (%)	27 (77%)	8 (23%)		

Table S3. Primers used for relative quantification of MACC1 SCNAs.

Gene	Primer position	Primer sequence
MACC1, amplicon 1	38345	TCCCTTCCCTGGAGAATCTT
	38450	TGCAGCTGATGATTCTGACC
MACC1, amplicon 2	5368	CACAGGGATATAACAGGAAGCTG
	5572	AGGCAGGTTTCCACATCATC
GATB1	511	AGTCAGAGCAAAAGGCGGTA
	632	AGGGGAAGAGGAAAGATGGA
NEURL4	187	AAGAAAGCAAGCCACTTGGA
	310	CAGCCTGTCCCTACTGCTTC

Table S4. MACC1 expression levels and alterations in key cancer genes.

Gene symbol	Mean *MACC1* expression in mutated samples	Mean *MACC1* expression in wild-type samples	Median *MACC1* expression in mutated samples	Median *MACC1* expression in wild-type samples	Wilcoxon *p* value
KRAS	10.37	10.62	10.52	10.76	0.005
POLE	9.85	10.53	10.02	10.61	0.042
HRAS	9.88	10.49	10.06	10.58	0.133
TP53	10.5	10.38	10.62	10.43	0.138
TGFBR2	10.07	10.52	10.37	10.59	0.139
MLH1	10.36	10.52	10.43	10.64	0.165
PTEN	10.51	10.38	10.62	10.52	0.249
SMAD4	10.4	10.56	10.56	10.58	0.316
BRAF	10.48	10.48	10.57	10.58	0.575
NRAS	10.56	10.47	10.77	10.55	0.618
APC	10.48	10.43	10.58	10.59	0.769
PIK3CA	10.47	10.5	10.57	10.64	0.868

Table S5. Multivariate Cox analysis including MACC1 expression, tumor size, stage and nodal status.

	Factor	DFS			OS		
		N	HR [CI]	*p* value	N	HR [CI]	*p* value
**Multivariate**	*MACC1* expression (High vs. Low)	154 - 167	1.69 [1.10 – 2.60]	0.02	181 - 185	1.44 [0.92– 2.55]	0.11
	T3–T4 vs. T1–T2	264 - 57	2.70 [1.06 – 6.87]	0.03	302 - 64	1.21 [0.53 – 2.77]	0.65
	Ns vs. N0	139 - 182	1.02 [0.31 – 3.30]	0.97	165 - 201	0.46 [0.18 – 1.20]	0.11
	Stage: III–IV vs. I–II	148 - 173	2.09 [0.62 – 6.99]	0.23	177 - 189	5.44 [1.98 – 14.93]	0.001
